# Toward elucidating diversity of neural mechanisms underlying insect learning

**DOI:** 10.1186/s40851-014-0008-6

**Published:** 2015-02-10

**Authors:** Makoto Mizunami, Yoshitaka Hamanaka, Hiroshi Nishino

**Affiliations:** Faculty of Science, Hokkaido University, Kita 10 Nishi 8, Kita-Ku, Sapporo, 060-0810 Japan; Research Institute for Electronic Science, Hokkaido University, Kita 12 Nishi 7, Kita-ku, Sapporo, 060-0811 Japan

**Keywords:** Crickets, Insects, Olfactory learning, Octopamine, Dopamine, Long-term memory, Evolution

## Abstract

Insects are widely used as models to study neural mechanisms of learning and memory. Our recent studies on crickets, together with reports on other insect species, suggest that some fundamental differences exist in neural and molecular mechanisms of learning and memory among different species of insects, particularly between crickets and fruit flies. First, we suggested that in crickets octopamine (OA) and dopamine (DA) neurons convey reward and punishment signals, respectively, in associated learning. On the other hand, it has been reported that in fruit flies different sets of DA neurons convey reward or punishment signals. Secondly, we have suggested that in crickets OA and DA neurons participate in the retrieval of appetitive and aversive memories, respectively, while this is not the case in fruit flies. Thirdly, cyclic AMP signaling is critical for short-term memory formation in fruit flies, but not in crickets. Finally, nitric oxide-cyclic GMP signaling and calcium-calmodulin signaling are critical for long-term memory (LTM) formation in crickets, but such roles have not been reported in fruit flies. Not all of these differences can be ascribed to different experimental methods used in studies. We thus suggest that there are unexpected diversities in basic mechanisms of learning and memory among different insect species, especially between crickets and fruit flies. Studies on a larger number of insect species will help clarify the diversity of learning and memory mechanisms in relation to functional adaptation to the environment and evolutionary history.

## Introduction

Insects have excellent learning and memory capabilities despite the relative simplicity of their central nervous systems, and thus they have been used as models to study basic mechanisms underlying learning and memory [1-5]. Much knowledge has been accumulated regarding neural and molecular mechanisms of learning and memory in a few species of insects, such as the fruit fly *Drosophila melanogaster*, the honeybee *Apis mellifera*, and the cricket *Gryllus bimaculatus*. In our studies in crickets, we noted that some of the basic features of neural mechanisms of learning and memory in crickets differ from those reported in fruit flies, although they are similar to those reported in honeybees. Such differences among insects have not been recognized in previous studies.

Here we review our major findings on learning and memory in crickets, and discuss how they are similar or different from those reported in other species. We also discuss whether the observed differences reflect species-specific features or can be explained by other factors such as the different experimental methods used. Finally, we briefly discuss the possible evolutionary perspective on diversity in learning and memory mechanisms in insects.

## Review

### Procedures for conditioning

**Figure 1:**
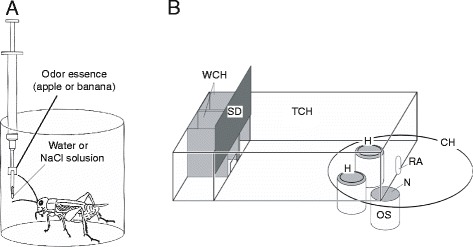
**Procedures for olfactory conditioning in crickets. (A)** For appetitive or aversive conditioning, an odor (e.g., apple or banana odor) was paired with water (appetitive US) or 20% sodium chloride solution (aversive US). A syringe containing water or sodium chloride solution was used for US delivery. A filter paper soaked with apple or banana essence was attached to the needle of the syringe. The filter paper was approached to the cricket’s antennae, and then water or sodium chloride solution was presented to the mouth. **(B)** Apparatus for the odor preference test. Two holes (H) connecting the chamber with odor sources (OS) were inserted in the floor of the test chamber (TCH). Each odor source consisted of a container with a filter paper soaked with apple or banana essence, covered with a fine gauze net (N). Three containers were mounted on a rotating container holder (CH), and two of three odor sources could be presented at the same time. A cricket was placed in the waiting chamber (WCH) for 4 min for acclimation and then allowed to enter the test chamber to visit odor sources, by opening a sliding door (SD). Two minutes later, the relative positions of the apple and banana sources were changed. The preference test lasted for 4 min. RA: rotating axle. Modified from Matsumoto and Mizunami [6].

We first briefly describe the experimental procedure used in our study of classical conditioning in crickets. We used a “classical conditioning and operant testing procedure”, which is based on the transfer of memory formed during classical conditioning to an operant testing situation [6,7]. For conditioning, crickets were individually placed in a beaker (Figure 1A). A filter paper soaked with an odor (conditioned stimulus, CS) was presented to the antennae, and then a drop of water or 20% sodium chloride solution (appetitive or aversive unconditioned stimulus, US) was applied to the mouth. In the operant odor preference test, crickets were individually placed in a test chamber and allowed to freely visit two odor sources on the floor (Figure 1B). The time that the crickets spent exploring each odor source with either the mouth or palpi was measured to evaluate relative odor preference of the crickets. In most experiments, crickets were subjected to an absolute appetitive or aversive conditioning procedure, in which an odor (CS+ or CS–) was paired with appetitive US or aversive US, and their preferences between the CS and a control odor were tested before and after conditioning. In other experiments, we used a differential conditioning procedure, in which an odor (CS+) was paired with appetitive US and another odor (CS–) was paired with aversive US. For pharmacological analysis, drugs were dissolved in cricket saline and injected into the hemolymph at 30 min before conditioning. Both within-group and between-group comparisons were used to evaluate the conditioning effect. Preferences before and after conditioning of a given group, as well as preferences after conditioning of different groups, were statistically compared.

**Figure 2:**
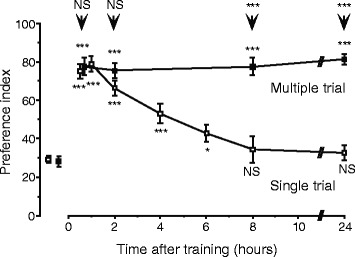
**Retention scores after single- and multiple-trial olfactory conditioning.** Seven groups of animals were subjected to single-trial conditioning (open squares) to associate a peppermint odor with water reward. Another four groups were subjected to two appetitive conditioning trials and two aversive conditioning trials, in which peppermint odor was paired with water and vanilla odor was paired with sodium chloride solution for two times each with an ITI of 5 min (black squares). Relative preferences of the peppermint odor (rewarded odor) compared with vanilla odor (control odor) were tested before and after conditioning in all groups. They were measured as preference indexes (PIs) and shown as mean ± SE. To simplify the figure, the PIs for rewarded odor ware shown as pooled data from seven single-trial or four multiple-trial groups. Preferences at each time after conditioning were compared to those before conditioning in each group (Wilcoxon’s test) and preferences of multiple-trial groups were also compared to those of single-trial groups at each time after conditioning (Mann-Whitney test), and the results of the former comparison are shown at each data point and those of the latter are shown *above* the arrow (*P < 0.05; **P < 0.01; ***P < 0.001; NS *P* < 0.05). The preferences for rewarded odor remained unchanged from 30 min to 24 hours after conditioning in the multiple-trial group (*P* < 0.05, Mann-Whitney test). Modified from Matsumoto et al. [32].

Crickets exhibited excellent olfactory learning capabilities. One appetitive or aversive conditioning trial led to memory that lasted for at most a few hours, which is insensitive to amnestic treatment and to protein synthesis inhibitors, and is characterized as mid-term memory (MTM). Multiple conditioning trials led to memory that lasted for at least 24 hours, which requires *de novo* protein synthesis and is characterized as long-term memory (LTM) (Figure 2) [6,8]. The excellent olfactory learning capabilities of crickets are evidenced by our findings that (1) training on three consecutive days on fourth-instar nymphal crickets leads to lifetime memory retention, which is readily rewritten in response to new experience [9], (2) crickets can memorize seven pairs of odors at the same time [10], and (3) crickets can exhibit some forms of higher-order learning, including context-dependent discrimination learning [11], second-order conditioning [12], and sensory preconditioning [13].

Studies of classical conditioning on fruit flies and honeybees have been performed using somewhat different procedures. In aversive classical conditioning in the fruit fly *Drosophila*, a group of flies was exposed to two odors in the training chamber, one of which is paired with electric shock and the other is not. The animals were then exposed to both odors in a T-maze, one odor from each side [14]. For appetitive conditioning, a group of flies was exposed to an odor during presentation of sucrose solution and then exposed to another odor without presentation of sucrose [15]. In appetitive conditioning in honeybees, a bee was placed in a metal tube and an odor was presented to the antennae and then sucrose solution was presented to the antennae and the mouth. After training, the bee extended its proboscis in response to the presentation of conditioned odor [2]. For aversive conditioning, a harnessed bee was presented with an odor and then electric shock. After training, the bee exhibited sting extension in response to the conditioned odor [16]. On account of differences in conditioning procedures, previous studies concluded that many features of learning and memory are much the same among different insects. Here we focus on our recent findings that do not support the general notion that basic features of learning and memory systems are much the same among insects.

### Roles of octopamine neurons and dopamine neurons in conveying reward and punishment signals

**Figure 3:**
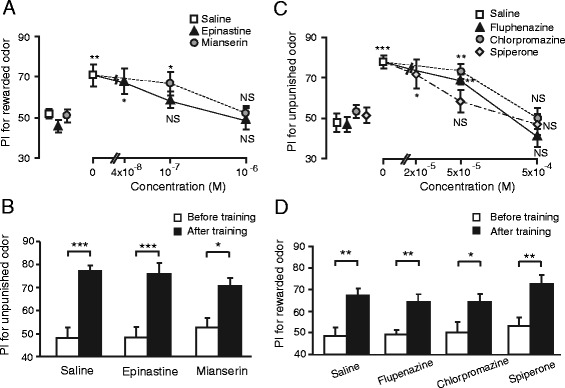
**Effects of OA or DA receptor antagonists on appetitive and aversive olfactory conditioning. (A)** Dose-dependent effects of OA receptor antagonists on appetitive olfactory conditioning. Six groups of crickets were injected with 3 μl saline (white squares) or saline containing 0.04 μM, 0.1 μM or 1 μM epinastine (black triangles) or 0.1 μM or 1 μM mianserin (gray circles). **(B)** Effects of OA receptor antagonists on aversive olfactory conditioning. Three groups of crickets were injected with 3 μl saline or saline containing 1 μM epinastine or 1 μM mianserin 30 min before 6-trial aversive conditioning. **(C)** Dose-dependent effects of DA receptor antagonists on aversive olfactory conditioning. Eight groups of crickets were injected with 3 μl saline (white squares) or saline containing 50 μM or 500 μM fluphenazine (black triangles), 50 μM or 500 μM chlorpromazine (gray circles) or 20 μM, 50 μM or 500 μM spiperone (white diamonds). **(D)** Effects of DA receptor antagonists on appetitive olfactory conditioning. Four groups of crickets were injected with 3 μl saline or saline containing 500 μM fluphenazine, 500 μM chlorpromazine or 500 μM spiperone 30 min before 2-trial aversive conditioning. Relative odor preferences were measured as preference indexes (PIs) for rewarded odor **(A, D)** or unpunished control odor **(B, C)** before (data points at the left) and at 30 min after conditioning (data points at the right) and are shown with means ± SEM. The results of statistical comparison before and after conditioning are shown as asterisks (Wilcoxon’s test, *P < 0.05; **P < 0.01; ***P < 0.001, NS *P* < 0.05). Modified from Unoki et al. [7].

Elucidation of the neural mechanisms mediating reward or punishment signals in learning is one of the major subjects in neuroscience. In mammals, dopamine (DA) neurons in the midbrain mediate reward and punishment signals in associative learning [17]. We studied the effects of octopamine (OA) and DA receptor antagonists on appetitive and aversive olfactory conditioning in crickets [7]. Crickets injected with an OA receptor antagonist (epinastine or mianserin) into the hemolymph before conditioning exhibited impairment of appetitive conditioning of an odor with water reward (Figure 3A). On the other hand, such crickets exhibited no impairment of aversive conditioning of the same odor with sodium chloride punishment (Figure 3B). Their responses to water reward were also intact. Thus, OA receptor antagonists do not impair perception of olfactory CS or gustatory US. We thus concluded that OA neurons are specifically involved in conveying water reward in conditioning. We also found that injection of a DA receptor antagonist (fluphenazine, chlorpromazine, or spiperone) completely impaired aversive conditioning of an odor with sodium chloride punishment (Figure 3C). In contrast, it did not impair appetitive conditioning of the odor with water reward or aversive response to sodium chloride solution, indicating that DA receptor antagonists do not impair sensory functions necessary for learning (Figure 3D). These results similarly suggest that DA neurons are specifically involved in conveying sodium chloride punishment in conditioning. We also studied the roles of OA and DA neurons in appetitive and aversive conditioning of a visual pattern [18] and a color cue [19] and obtained the same results.

These findings in crickets are consistent with those in honeybees. In bees, it has been reported that OA neurons play roles in appetitive olfactory conditioning with sucrose reward [20,21], whereas DA neurons play roles in aversive olfactory conditioning with electric shock [16].

In fruit flies, some early neurogenetic studies suggested that OA and DA neurons convey sucrose reward and electric shock punishment, respectively, in olfactory conditioning [15,22], but recent extended studies have revealed that different subsets of DA neurons projecting to the mushroom body convey reward or punishment signals in olfactory learning [23-27]. The mushroom body is a higher-order olfactory and multisensory center in the insect brain that is implicated in olfactory and other forms of learning [1,2,4,28,29,30]. In fruit flies, OA neurons have been shown to act upstream of DA neurons and send sweet taste signal to DA neurons in appetitive learning [26,27]. The critical difference between fruit flies and crickets, therefore, is that DA neurons play critical roles in appetitive learning in fruit flies, but not in crickets. In fruit flies, it has been shown that appetitive reinforcement by DA neurons is mediated by Type 1 dopamine receptor (DopR1).

It can be argued that the observed difference in the roles of DA neurons in appetitive learning may be attributed to the difference of appetitive US used in the experiments; namely, water was reward in crickets whereas sucrose reward was used in fruit flies. This argument, however, does not match the finding in honeybees that OA neurons mediate sucrose reward in appetitive learning [20,21].

### Roles of octopamine neurons and dopamine neurons in appetitive and aversive memory retrieval

**Figure 4:**
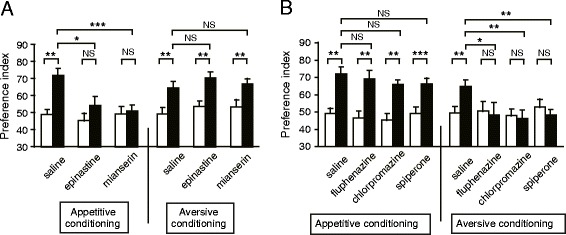
**Effects of OA (A) and DA (B) receptor antagonists on olfactory memory retrieval.** Twelve groups of crickets were each subjected to 2-trial appetitive (left) or 6-trial aversive (right) olfactory conditioning trials. On the next day, each group was injected with 3 μl of saline or saline containing 1 μM epinastine, 1 μM mianserin, 500 μM fluphenazine, 500 μM chlorpromazine or 500 μM spiperone at 30 min before the final test. Preference indexes for the rewarded odor (in the case of appetitive conditioning) or unpunished control odor (in the case of aversive conditioning) before (white bars) and one day after (black bars) conditioning are shown with means + SEM. The results of statistical comparison before and after conditioning (Wilcoxon’s test) and between experimental and saline-injected control groups (Mann-Whitney test) are shown as asterisks (*P < 0.05; **P < 0.01; ***P < 0.001, NS *P* < 0.05). Modified from Mizunami et al. [12].

We also found that OA and DA neurons participate in appetitive and aversive memory retrieval, respectively, in crickets [12]. Crickets were subjected to appetitive or aversive olfactory conditioning and were injected with an OA or DA receptor antagonist before a retention test. Injection of an OA receptor antagonist (epinastine or mianserin) impaired appetitive olfactory memory retrieval, but had no effect on aversive olfactory memory retrieval (Figure 4A). On the other hand, injection of a DA receptor antagonist (fluphenazine, chlorpromazine, or spiperone) impaired aversive memory retrieval but had no effect on appetitive memory retrieval (Figure 4B). We also found that injection of OA and DA receptor antagonists impaired appetitive and aversive memory retrieval, respectively, in visual pattern conditioning [12]. Therefore we concluded that OA and DA neurons participate in the retrieval of appetitive memory and aversive memory, respectively, in both olfactory and visual pattern learning.

This is in accordance with finding in honeybees that disruption of OA-ergic transmission in the antennal lobe, the primary olfactory center, by an OA receptor antagonist (mianserin) or by RNAi of the OA receptor gene disrupted appetitive olfactory memory retrieval [21]. The possible roles of DA neurons in aversive memory retrieval, however, have not been tested in honeybees.

In fruit flies, DA neurons participate in formation, but not retrieval, of electric shock-induced aversive memory [15]. In addition, it has been concluded that a subset of DA neurons projecting to the mushroom body (called PAM neurons) participates in formation, but not retrieval, of sugar-induced appetitive memory [24]. Thus, it appears that OA and DA neurons participate in memory retrieve in crickets but not in fruit flies. We discuss the implications of these findings in the next section.

### Models of classical conditioning in insects

**Figure 5:**
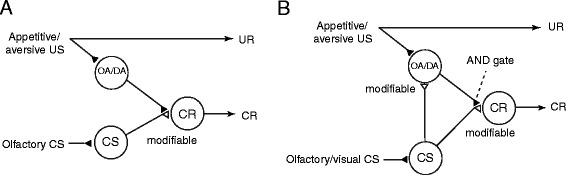
**Conventional model and our model of classical conditioning in insects. (A)** A model proposed to account for the roles of intrinsic and extrinsic neurons of the mushroom body in olfactory conditioning in fruit flies [15]. OA neurons and DA neurons (“OA/DA” neurons) convey signals for appetitive and aversive US, respectively. “CS” neurons, which convey signals for CS, make synaptic connections with “CR” neurons that induce a conditioned response (CR), the efficacy of the connection being strengthened by conditioning (open triangles, marked as modifiable). “OA/DA” neurons make synaptic connections with axon terminals of “CS” neurons. **(B)** Our model proposed to account for classical conditioning in crickets. The model assumes that efficacy of synaptic transmission from “CS” neurons to “OA/DA” neurons is strengthened by conditioning and that coincident activation of “OA/DA” neurons (open triangles, marked as modifiable) and simultaneous activation of “CS” neurons and “OA/DA” neurons is needed to activate “CR” neurons to lead to a CR (AND gate). No synaptic plasticity is assumed for synapses with filled triangles. Modified from Mizunami et al. [12].

Because our findings in crickets that OA or DA neurons participate in appetitive or aversive memory retrieval, respectively, were not consistent with conventional neural models of classical conditioning proposed in fruit flies [15], we propose a new model [12]. Figure 5A depicts a model proposed by Schwaerzel et al. [15] to account for the roles of extrinsic and intrinsic neurons of the mushroom body in appetitive or aversive olfactory conditioning with sucrose reward or electric shock punishment in the fruit fly. The neural events that this model assumes are as follows: (1) “CS” neurons (intrinsic neurons comprising the mushroom body, called Kenyon cells) that convey signals about a CS make synaptic connections with dendrites of “CR” neurons (efferent neurons of the mushroom body lobe), activation of which leads to a conditioned response (CR) that mimics an unconditioned response (UR), but these synaptic connections are silent or very weak before conditioning, (2) OA- and DA-ergic efferent neurons projecting to the lobes (“OA/DA” neurons), which convey signals for appetitive and aversive US, respectively, make synaptic connections with axon terminals of “CS” neurons, and (3) the efficacy of synaptic transmission from “CS” neurons to “CR” neurons that induces a conditioned response (“CS–CR” synapse, which might not be monosynaptic) is strengthened by coincident activation of “CS” neurons and “OA/DA” neurons during conditioning.

We have proposed a new model (Figure 5B), with minimal modifications to the previous one by Schwaerzel et al. [15]. In our model, we assumed that (1) co-activation of “OA/DA” neurons and “CS” neurons are needed to activate “CR” neurons after conditioning (AND gate) and (2) synaptic connection from “CS” neurons to “OA/DA” neurons representing US (“CS–OA/DA” synapse) is strengthened by coincident activation of “CS” neurons and “OA/DA” neurons by pairing of a CS with a US. Strengthening of this synapse allows activation of “OA/DA” neurons by CS presentation and by subsequent activation of “CS” neurons, which then allows activation of “CR” neurons. In short, our model assumes enhancement of two synapses, “CS-CR” synapses and “CS-OA/DA” synapses, by conditioning; namely, it assumes formation of multiple memory traces [12,31]. It also assumes Kandelian synaptic plasticity for “CS–CR” synapses and Hebbian plasticity for “CS–OA/DA” synapses.

We also showed participation of OA and DA neurons in appetitive and aversive forms of second-order conditioning [12] and sensory preconditioning [13], and showed that these high-order learning phenomena can be accounted for by our new models with some modification of the above model.

### Roles of cyclic AMP signaling in formation of short-term memory

In fruit flies, cyclic AMP (cAMP) signaling plays critical roles in formation of olfactory short-term memory (STM), a memory phase that lasts a few minutes after conditioning and is sensitive to amnestic treatment [4]. For example, *rutabaga* mutants, with defects in adenylyl cyclase, an enzyme producing cAMP, and *dunce* mutants, with defects in phosphodiesterase (PDE), which degrades cAMP, both exhibit deficiency in STM. In crickets, on the other hand, pharmacological intervention of cAMP signaling by an inhibitor of adenylyl cyclase or cAMP-dependent protein kinase (PKA) impairs formation of LTM but neither STM nor MTM [32]. A recent study in honeybees also showed that inhibitors of adenylyl cyclase do not block STM and MTM [33], indicating that biochemical processes underlying STM in crickets and honeybees differ from those in fruit flies.

It has been shown that *rutabaga* mutants exhibit a low but significant level of olfactory STM, and thus fruit flies possess a cAMP-independent component of STM [4]. Whether this minor component of STM in flies is based on biochemical processes similar to those underlying STM in crickets and honeybees remains a subject for future studies.

### Roles of nitric oxide-cyclic GMP signaling in formation of long-term memory

**Figure 6:**
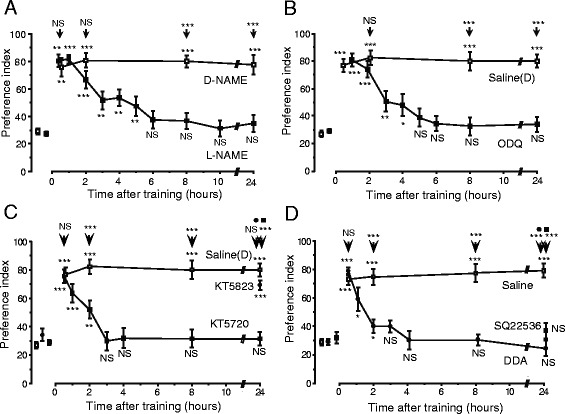
**Effects of inhibitors of components of the NO-cGMP signaling pathway (A and B) and cAMP signaling pathway (C and D) on olfactory LTM formation.** At 20 min prior to multiple-trial conditioning, animals were each injected with 3 μl of saline or saline containing various drugs. The preference indexes (PIs) before and at various times after conditioning are shown as means ± S. E. In **(A)**, animals in ten experimental groups were each injected with the NOS inhibitor L-NAME (400 μM) (black squares) and animals in another four control groups were each injected with the inactive isomer D-NAME (400 μM) (open squares). In **(B)**, animals in twelve groups were each injected with the sGC inhibitor ODQ (200 μM) dissolved in saline containing 0.1% DMSO (black squares), or saline containing 0.1% DMSO (saline (D) group, open squares). In **(C)**, animals in twelve groups were each injected with the PKA inhibitor KT5720 (200 μM) (black squares), the cGMP-dependent protein kinase (PKG) inhibitor KT5823 (1 mM) (black circles), or saline containing 0.1% DMSO (open squares). In **D**, animals in twelve groups were each injected with the adenylyl cyclase inhibitor DDA (1 mM) (black circles), another adenylyl cyclase inhibitor, SQ22536 (1 mM) (black squares), or saline (open squares). PIs before conditioning are shown as pooled data from all experimental or control groups. Odor preferences were compared before and after conditioning for each group (Wilcoxon’s test) and between experimental and control groups at each time after conditioning (Mann-Whitney test), and the results are shown at each data point and above the arrow, respectively (*P < 0.05; **P < 0.01; ***P < 0.001; NS *P* < 0.05). Modified from Matsumoto et al. [32].

**Figure 7:**
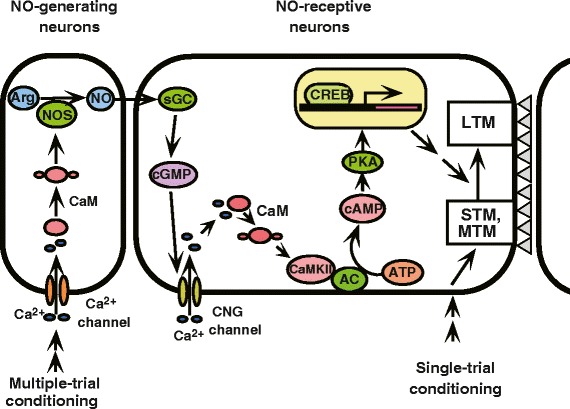
**A model proposed to account for the signaling cascade underlying LTM formation in crickets.** Single-trial conditioning induces only short-term synaptic plasticity that underlies amnesic treatment-sensitive short-term memory (STM) and amnesic-treatment resistant mid-term memory (MTM). Multiple-trial conditioning activates the NO-cGMP system, and this in turn activates adenylyl cyclase (AC) and then PKA via the cyclic nucleotide-gated (CNG) channel, calcium-calmodulin (CAM) system and CaM kinase II (CaMKII). Activation of PKA is assumed to activate a transcription factor, cAMP-responsive element-binding protein (CREB), which leads to protein synthesis that is necessary to achieve long-term plasticity of synaptic connection (a column of gray triangles) to other neurons assumed to be necessary for LTM. Arg: arginine, NOS: NO synthase, sGC: soluble guanylyl cyclase. We speculate that outer Kenyon cells generate NO and inner Kenyon cells receive NO to achieve long-term synaptic plasticity for LTM formation [38]. Modified from Mizunami et al. [36].

Nitric oxide (NO) is a membrane-permeable molecule that functions in intercellular signaling [34]. It is produced by NO synthase (NOS), diffuses into neighboring cells, and stimulates soluble guanylyl cyclase (sGC) to produce cyclic GMP (cGMP) [34]. We found that injection of inhibitors of the enzyme catalyzing the formation of NO, cGMP, or cAMP prior to multiple-trial conditioning blocks LTM (Figure 6), whereas injection of an NO donor, a cGMP analogue, or a cAMP analogue prior to single-trial conditioning induces LTM. These observations suggest that the NO-cGMP pathway and cAMP pathway participate in LTM formation [32,35]. LTM induced by injection of an NO donor or a cGMP analogue paired with single-trial conditioning was blocked by inhibition of the cAMP pathway, but induction of LTM by a cAMP analogue was unaffected by inhibition of the NO-cGMP pathway, suggesting that the cAMP pathway is a downstream target of the NO-cGMP pathway for LTM formation. Inhibitors of the cyclic nucleotide-gated channel (CNG channel) or calmodulin blocked induction of LTM by the cGMP analogue paired with single-trial conditioning, but they did not affect induction of LTM by the cAMP analogue. Moreover, we recently obtained evidence to suggest that CaMKII intervenes between calmodulin and adenylyl cyclase, an enzyme producing cAMP [36]. These results suggest that the CNG channel and calcium-calmodulin and CaMKII are downstream targets of the NO-cGMP pathway and are upstream of the cAMP pathway (Figure 7). We also found that NO-cGMP signaling plays a critical role in LTM formation in visual learning in crickets [37].

We also demonstrated that RNAi of the *NOS* gene impairs olfactory LTM formation in crickets [38]. In situ hybridization demonstrated a high level of *NOS* mRNA expression in outer Kenyon cells of the mushroom body, in addition to some neurons around the antennal lobe and the base of the optic lobe. We thus assume that olfactory LTM is formed in the mushroom body, by interaction of outer and inner Kenyon cells.

We also demonstrated participation of NO signaling in LTM formation in cockroaches. Cockroaches exhibit an increased level of salivation in response to an odor paired with sucrose reward [39], which can be monitored by changes in responses of salivary neurons to odors [40,41]. Injection of an NOS inhibitor impairs formation of LTM, but not that of STM or MTM, in olfactory conditioning of activities of salivary neurons [42].

Studies in honeybees also suggested that the NO-cGMP signaling pathway and cAMP pathway in the antennal lobe act in parallel and are complementary for the formation of LTM [43,44]. This is in contrast to our conclusion that the NO-cGMP system and the cAMP system are serially arranged for LTM formation in crickets [35]. Thus, the manner by which NO signaling contributes to LTM formation may not be identical between crickets and honeybees. Participation of the CNG channel, calcium-calmodulin and CaMKII in LTM formation has also been demonstrated in honeybees [42].

Interestingly, so far no report has suggested the involvement of NO-cGMP signaling, or calcium-calmodulin signaling in biochemical cascades underlying LTM formation in fruit flies, although there have been extensive studies on biochemical cascades underlying LTM formation [4]. Biochemical cascades for LTM formation in fruit flies may differ from those in crickets and honeybees.

### Evolutionary considerations

The findings discussed in this review, summarized in Table 1, suggest that many features of cellular and biochemical processes underlying learning and memory in fruit flies differ from those in crickets and honeybees. In contrast, the features of neural processes underlying learning and memory in honeybees are similar to those in crickets (Table 1). Crickets belong to an evolutionary basal group (the order Orthoptera), and fruit flies and honeybees belong to the order Diptera and Hymenoptera, respectively, both of which have emerged more recently. If the unique features of learning and memory in fruit flies represent those of dipteran insects, they may be due to re-organization of their central nervous system during the course of evolution. Dipterans are highly adapted for rapid aerial movement. They have short and streamlined bodies and large compound eyes, the hindwings are changed to halteres that function as gyroscopes, the mechanoreceptors of which inform the insect about rotation of the body during flight, and the brain is characterized by a large optic lobe (visual center) and a small mushroom body. Learning and memory mechanisms of dipterans may have been simplified as a trade-off to develop sophisticated neural mechanisms for visual and mechanosensory control of swift movement in the air. On the other hand, honeybees and other hymenopteran insects posses a high capability to learn the location, color, shape and odor of foods [2] and thus they may have retained, or elaborated, learning and memory systems established in earlier stages of insect evolution. Comparisons of only three species, however, do not allow deeper discussion on evolution, and studies on of a larger number of species, including apterygotes, are needed for better understanding of commonalities and diversities of learning and memory systems among insects.

**Table 1:** **Comparison of neural processing underlying learning in three insect species**

	**Crickets (** ***Gryllus bimaculatus*** **)**	**Fruit flies (** ***Drosophila melanogaster*** **)**	**Honeybees (** ***Apis mellifera*** **) (A or B?)**
Learning	A: OA and DA neurons mediate reward and punishment signals, respectively [7,18,19].	B: DA neurons mediate both reward and punishment signals; OA neurons send reward signal to DA neurons in appetitive learning [23,24,26].	A [16,20,21]
Memory retrieval	A: OA and DA neurons participate in appetitive and aversive memory retrieval, respectively [12].	B: OA or DA neurons do not participate in memory retrieval [15,24].	A [21]
STM formation	A: cAMP signaling does not participate in STM formation [32,35].	B: cAMP signaling participates in STM formation [4].	A [33]
LTM formation	A: NO-cGMP signaling participates in LTM formation [32,35].	B: There is no report on participation of NO-cGMP signaling in LTM formation.	A [43,44]

LTM: long-term memory: STM: short-term memory.

## Conclusion

We conclude that there are unexpected diversities in basic mechanisms of learning and memory among different insect species, especially between crickets and fruit-flies. We recently found that the discrepancy, or error, between the actual US and the predicted US determines whether learning occurs in crickets [45], indicating that the prediction error theory is applicable to crickets. It is of great significance to study whether this finding is applicable to other species of insects. Insects are one of the most successful animals group in terms of species richness and diversity of life styles [46,47]. Elucidation of diversity of learning and memory mechanisms in insects in relation to functional adaptation to the environments and evolutionary history should emerge as a fascinating future subject.

## Abbreviations

OA: Octopamine
DA: Dopamine
cAMP: Cyclic adenosine monophosphate
cGMP: Cyclic guanosine monophosphate
LTM: Long-term memory
MTM: Mid-term memory
STM: Short-term memory
US: Unconditioned stimulus
CS: Conditioned stimulus
UR: Unconditioned response
CR: Conditioned response
DopR1: Type 1 dopamine receptor
PDE: Phosphodiesterase
PKA: Cyclic AMP-dependent protein kinase
NO: Nitric oxide
CNG channel: Cyclic nucleotide-gated channel
CaMKII: Calcium/calmodulin-dependent kinase II
NOS: NO synthase
sGC: Soluble guanylyl cyclase
CREB: Cyclic AMP-responsive element-binding protein
